# Network meta-analysis of mineralocorticoid receptor antagonists for diabetic kidney disease

**DOI:** 10.3389/fphar.2022.967317

**Published:** 2022-09-16

**Authors:** Yichuan Wu, Huanjia Lin, Yuan Tao, Ying Xu, Jiaqi Chen, Yijie Jia, Zongji Zheng

**Affiliations:** ^1^ Department of Endocrinology and Metabolism, Nanfang Hospital, Southern Medical University, Guangzhou, China; ^2^ De Feng Academy, Southern Medical University, Guangzhou, China

**Keywords:** diabetic kidney disease (DKD), mineralocorticoid receptor antagonists (MRA), type 2 diabetes, hyperkalemia, network meta-analysis (NMA)

## Abstract

Diabetic kidney disease (DKD) is one of the major causes of end-stage renal disease (ESRD). To evaluate the efficacy and safety of different types of mineralocorticoid receptor antagonists (MRAs) in diabetic kidney disease patients, we conducted this network meta-analysis by performing a systematic search in PubMed, MEDLINE, EMBASE, Web of Science, the Cochrane Library, and Clinicaltrials.gov. A total of 12 randomized clinical trials with 15,492 patients applying various types of MRAs covering spironolactone, eplerenone, finerenone, esaxerenone, and apararenone were included. The efficacy outcomes were the ratio of urine albumin creatine ratio (UACR) at posttreatment vs. at baseline, change in posttreatment estimated glomerular filtration (eGFR) vs. at baseline, and change in posttreatment systolic blood pressure (SBP) vs. at baseline. The safety outcome was the number of patients suffering from hyperkalemia. High-dose finerenone (MD −0.31, 95% CI: −0.52, −0.11), esaxerenone (MD −0.54, 95% CI: −0.72, −0.30), and apararenone (MD −0.63, 95% CI: −0.90, −0.35) were associated with a superior reduction in proteinuria in patients with DKD. Regarding the change in eGFR, the results of all drugs were similar, and finerenone may have potential superiority in protecting the kidney. Compared with placebo, none of the treatments was associated with a higher probability of controlling systolic blood pressure during treatment. Moreover, spironolactone, esaxerenone, and 20 mg of finerenone presented a higher risk of hyperkalemia. This Bayesian network meta-analysis was the first to explore the optimal alternative among MRAs in the treatment of DKD and revealed the superiority of 20 mg of finerenone among MRAs in treating DKD.

**Systematic Review Registration:** PROSPERO, identifier (CRD42022313826)

## 1 Introduction

Diabetic kidney disease, or DKD, is a common type of complication among diabetes patients, whose morbidity has risen sharply in recent years (Tuttle et al., 2014). Additionally, as the leading cause of end-stage renal disease (ESRD), DKD causes huge health and economic burdens for both patients and society (Johansen et al., 2021). Although the mechanism of diabetic kidney disease is still unclear, we and others have revealed that diabetic kidney disease may be related to renal fibrosis, among which the relationship between microRNA (miRNA) crosstalk and renal epithelial tubular cell epithelial-mesenchymal transition (EMT) and endothelial-to-mesenchymal transition (EndMT), leads to fibrosis in kidney. (Wang et al., 2014; Zheng et al., 2016; Jia et al., 2019; Srivastava et al., 2019; Wang et al., 2019; Ding et al., 2021; Zheng Y. et al., 2022). Moreover, sirtuins, which are a part of class III HDAC, are associated with various metabolic signs of progress, for example, aging, apoptosis, and inflammation. Studies have demonstrated that the suppression of SIRT3 protein in diabetic kidneys causes abnormal glycolysis, which is related to fibrosis in the kidney. Also, the SIRT3 protein in endothelial cells is linked with. (Liu et al., 2017; Srivastava et al., 2018).

Various treatments have been performed on DKD, while no single class of drugs has presented outstanding efficacy (Doshi and Friedman, 2017). Therefore, the combination of different kinds of drugs should be considered for managing DKD. Both albuminuria and decreased eGFR can be used for the prediction of DKD (Ninomiya et al., 2009), while albuminuria has been the most investigated sign of DKD in various forms, such as the urine albumin creatine ratio (UACR) (Mogensen, 1984). Studies have shown that DKD may be associated with the hyperactivation of the mineralocorticoid receptor (MR) (Jaisser and Farman, 2016). Mineralocorticoid receptor antagonist (MRA) has been proven to effectively protect the cardiovascular system and the kidney by competitively combining with MR (Draznin et al., 2022). Traditional MRAs include spironolactone and eplerenone; however, they are not widely used clinically owing to the high incidence of hyperkalemia (Bomback et al., 2008). Finerenone is a new nonsteroidal MRA with optimal efficacy and safety in treating patients with DKD (Draznin et al., 2022). As a selective nonsteroidal drug, esaxerenone has been proven to have antihypertensive and renal-protective effects (Jankovic and Jankovic, 2022). Apararenone was shown to protect renal functions in patients with DKD in a phase 2 study (Wada et al., 2021).

The existing evidence for the treatment of MRAs in DKD remains unclear (Draznin et al., 2022). Previous meta-analyses related to mineralocorticoid receptor antagonists were mostly about cardiovascular diseases (Yang et al., 2019). Chen et al. (2021) reported that for those who suffered from acute myocardial infarction, mineralocorticoid receptor antagonists reduced cardiovascular adverse events and all-cause mortality. Moreover, finerenone has been proven effective in treating patients with DKD (D'Marco et al., 2021). However, few trials have directly compared the efficacy and safety of MRAs in patients with DKD, and few studies have compared different kinds of mineralocorticoid receptor antagonists with placebo to compare their efficacy and safety outcomes. As an extension of conventional meta-analysis, network meta-analysis (NMA) can compare the results of a series of drugs for a disease simultaneously in the absence of head-to-head evidence to determine the best treatment.

We performed this systematic review and network meta-analysis of randomized clinical trials to compare both traditional and new MRAs in adults with diabetic kidney disease.

## 2 Methods

This study was conducted according to the PRISMA Extension Statement for Reporting of Systematic Reviews Incorporating Network Meta-analyses of Health Care Interventions: Checklist and Explanations. The protocol of this study was registered with PROSPERO (CRD42022313826).

### 2.1 Data sources and search strategies

This network meta-analysis was conducted to compare the effect and safety outcomes of five kinds of MRAs on clinical outcomes. We searched PubMed, MEDLINE, EMBASE, Web of Science, the Cochrane Library, and Clinicaltrials.gov from 1 January 2000, to 23 April 2022. The keywords utilized were as follows: “Diabetic Nephropathy,” “Diabetic Kidney Disease,” “Diabetic Nephropathies,” “Diabetic Glomerulosclerosis,” “DN,” “Kimmelstiel-Wilson Syndrome,” “DKD,” “nephropathy, diabetic,” “Mineralocorticoid Receptor Antagonist,” “MRA,” “Aldosterone Receptor Antagonist,” “antagonists, mineralocorticoid,” “antagonist, aldosterone receptor,” “spironolactone,” “finerenone,” “eplerenone,” “esaxerenone,” “apararenone,” “BAY 94-8862,” “MT-3995,” and “CS-3150.” References cited in identified papers and meta-analyses were reviewed in case of neglect. The details of the search strategy are listed in the Supplementary Table S1.

### 2.2 Study selection and data extraction

The articles were included if they met the inclusion criteria listed as follows: 1) the patients included in the study were adults suffering from T2DM and chronic kidney disease (CKD); 2) studies conducted as randomized clinical trials of treatment groups utilizing MRA and control group using placebo; 3) studies with outcomes of “UACR” or “eGFR” or “SBP” or “adverse event.”

Studies were excluded if 1) they were not randomized clinical trials, such as systemic reviews, comments, or case reports; 2) they focused on nonhuman subjects; 3) the studies only contained treatment groups or the control groups did not use placebo; or 4) they lacked basic data for analysis.

Two investigators independently searched articles and extracted data. Any disagreements concerning data were resolved with the third author. We extracted the name of the first author, year of publication, sample size, interventions, follow-up time, efficacy, and safety outcomes.

### 2.3 Efficacy and safety outcomes

The efficacy outcomes included the ratio of UACR at posttreatment vs. at baseline, change in posttreatment estimated glomerular filtration (eGFR) vs. at baseline, and change in posttreatment systolic blood pressure (SBP) vs. at baseline. The safety outcome was shown as the number of patients suffering from hyperkalemia. Posttreatment was defined as after the end of the administration of MRAs.

### 2.4 Risk of bias assessment

We used the Cochrane risk of bias tool to assess risk, including seven sections: random sequence generation, allocation concealment, blinding of personnel and participants, blinding of outcome assessment, selective reporting, method of addressing incomplete data, and other bias. For each part, studies were assessed to have a low, high, or unclear risk of bias. A graphic of bias was generated with Review Manager 5.4 (Cochrane Collaboration, Oxford, United Kingdom). Two authors independently assessed bias and any disagreements were resolved by consensus.

### 2.5 Statistical analysis

This Bayesian network meta-analysis was conducted by R software utilizing the “gemtc” package, which recalled JAGS in R for Markov chain Monte Carlo (MCMC) sampling. Data were analyzed using a random-effect model. Dichotomous outcomes were evaluated by a binomial likelihood model with a logit link function while continuous outcomes were calculated using a normal likelihood model with an identity link function. Risk ratios (RRs) and 95% credible intervals (95% CIs) were used to evaluate dichotomous outcomes, and for continuous outcomes, mean differences (MDs) and 95% CIs were used. Model convergence was assessed utilizing the Brooks–Gelman–Rubin statistic and trace plots. We generated 50,000 iterations for each analysis and discarded the first 10,000 iterations as a burn-in period. To rank each outcome, we utilized surface under the cumulative ranking area (SUCRA) probabilities. The larger the SUCRA was, the higher the probability of an endpoint event. Nodal analyses were used for heterogeneity checks. Over 50% of I^2^ indicated significant heterogeneity while I^2^ less than 50% indicated little heterogeneity.

## 3 Results

### 3.1 Baseline characteristics of the studies

**FIGURE 1:**
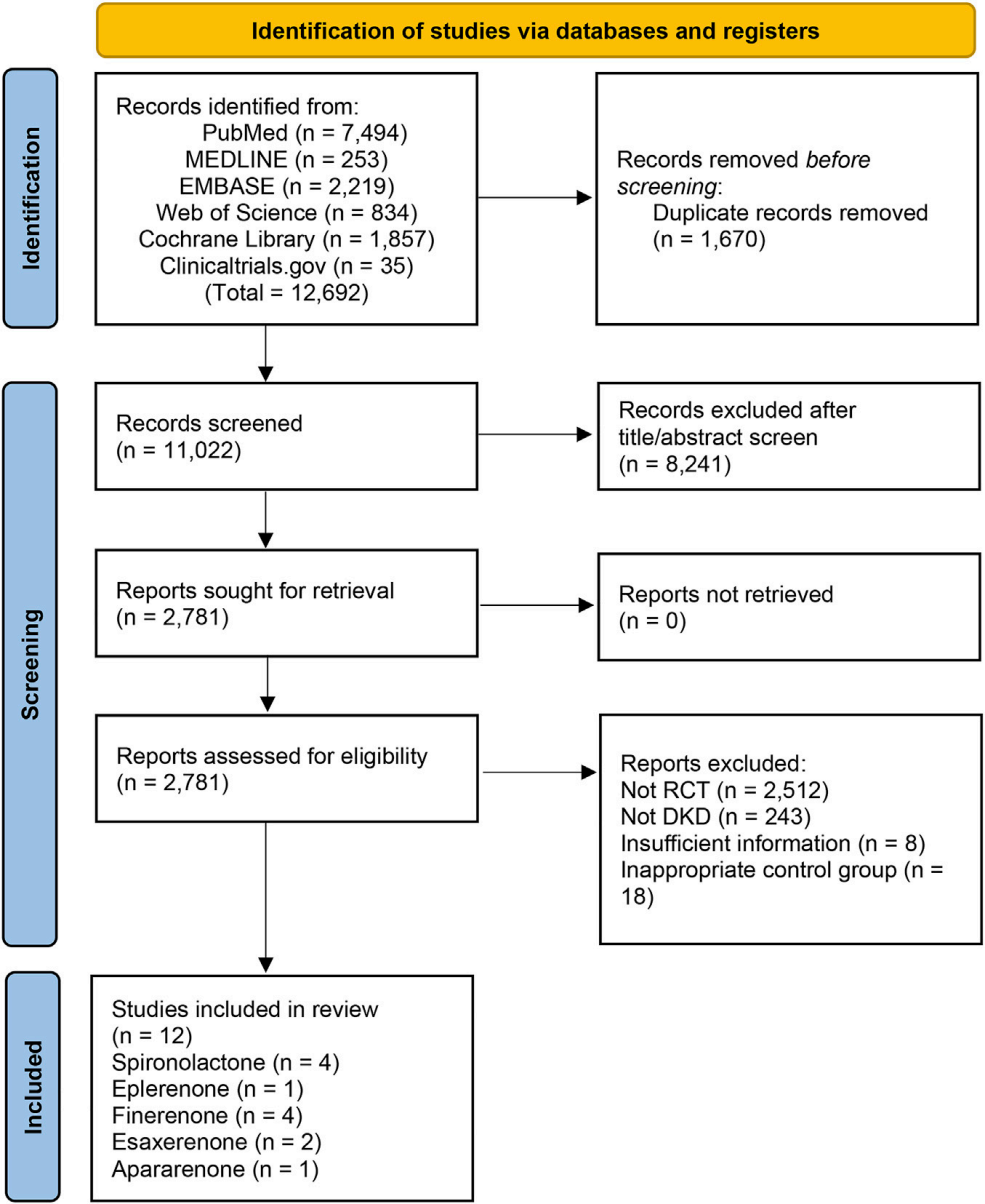
PRISMA flow diagram used for study selection.

A total of 12,692 studies were retrieved from the databases mentioned above, and 12 studies of 15,492 patients were eligible for our study. The details of the selection are shown in Figure 1. Spironolactone was utilized in four studies, one for eplerenone, four for finerenone, two for esaxerenone, and one for apararenone. The sample size of each study ranged from 35 to 7,352, while the duration of treatment varied from 12 to 152 weeks. Of all studies, 6 reported the ratio of UACR at posttreatment vs. at baseline, 6 provided data on change in posttreatment eGFR vs. at baseline, 5 submitted data on change in posttreatment SBP vs. at baseline, and 10 studies reported the morbidity of hyperkalemia. The overall details of the eligible studies are shown in Table 1.

**TABLE 1:** Baseline characteristics of eligible studies.

Study	Country	Age (T/C)	Sample size (T/C)	Intervention (T/C)	Duration of treatment	Duration of follow-up	Outcomes
Bakris 2015(Bakris et al., 2015)	United States	64.33 ± 9.21/63.26 ± 8.68	727/94	RAS blocker + Finerenone (1.25, 2.5, 5, 7.5, 10, 15, 20 mg)/RAS blocker + placebo	90 days	-	①②③④
Bakris 2020(Bakris et al., 2020)	United States	65.4 ± 8.9/65.7 ± 9.2	2,833/2,841	RAS blocker + Finerenone (10 mg, 20 mg)/RAS blocker + placebo	44 weeks	-	②④
Katayama 2017(Katayama et al., 2017)	Japan	62.40 ± 9.80/66.75 ± 9.02	84/12	RAS blocker + Finerenone (1.25, 2.5, 5, 7.5, 10, 15, 20 mg)/RAS blocker + placebo	90 days	30 days	①③④
Pitt 2021(Pitt et al., 2021)	United States	64.1 ± 9.7/64.1 ± 10.0	3,686/3,666	RAS blocker + Finerenone (10 mg, 20 mg)/RAS blocker + placebo	54 weeks	-	①②④
Epstein 2006(Epstein et al., 2006)	United States	60/58	177/91	Eplerenone (50 mg, 100 mg) + enalapril/placebo + enalapril	12 weeks	-	④
Ito 2019(Ito et al., 2019)	Japan	65.3 ± 9.3/66.0 ± 10.0	285/73	ACEi/ARB + Esaxerenone (0.625, 1.25, 2.5, 5 mg)/ACEi/ARB + placebo	12 weeks	6 weeks	①②④
Ito 2020(Ito et al., 2020b)	Japan	66 ± 10/66 ± 9	222/227	ACEi/ARB + Esaxerenone (1.25–2.5 mg)/ACEi/ARB + placebo	52 weeks	-	①④
Mehdi 2009(Mehdi et al., 2009)	United States	49.3 ± 8.8/51.7 ± 9.3	27/27	Lisinopril + Spironolactone (25 mg)/lisinopril + placebo	48 weeks	4 weeks	④
Wada 2021(Wada et al., 2021)	Japan	62.3 ± 9.0/60.1 ± 10.0	220/72	ACEi/ARB + Apararenone (2.5, 5, 10 mg)/ACEi/ARB + placebo	24 weeks	8 weeks	①②
Momeni 2015(Momeni et al., 2015)	Iran	58.9 ± 9.3/55.4 ± 8.9	20/20	Hydrochlorothiazide + Spironolactone (50 mg)/hydrochlorothiazide + placebo	12 weeks	-	③
van den Meiracker 2006(van den Meiracker et al., 2006)	Netherland	29-78	24/29	ACEi/ARB + Spironolactone (20–40 mg)/ACEi/ARB + placebo	52 weeks	-	②③④
Kota 2012(Kumar Kota et al., 2012)	India	45.6 ± 13.1/48.1 ± 12.5	19/16	ACEi/ARB + Spironolactone (25 mg)/ACEi/ARB + placebo	12 weeks	-	③④

Notes: T, treatment group; C, control group; -, Not mentioned; ①, ratio of UACR at posttreatment vs. at baseline; ②, change in posttreatment eGFR vs. at baseline; ③, change in posttreatment SBP vs. at baseline; ④, morbidity of hyperkalemia.

**FIGURE 2:**
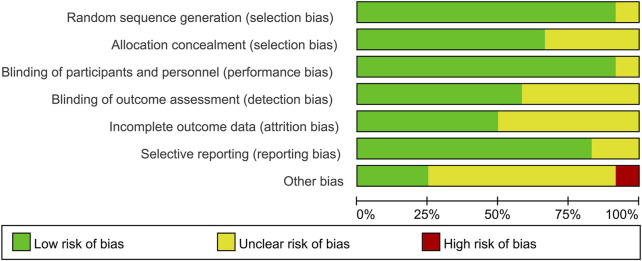
Risk of bias graph. The green symbols indicate for low risk of bias, the yellow symbols indicate for unclear risk of bias, and the red symbols indicate for high risk of bias. This figure was generated using Review Manager Version 5.4.

**FIGURE 3:**
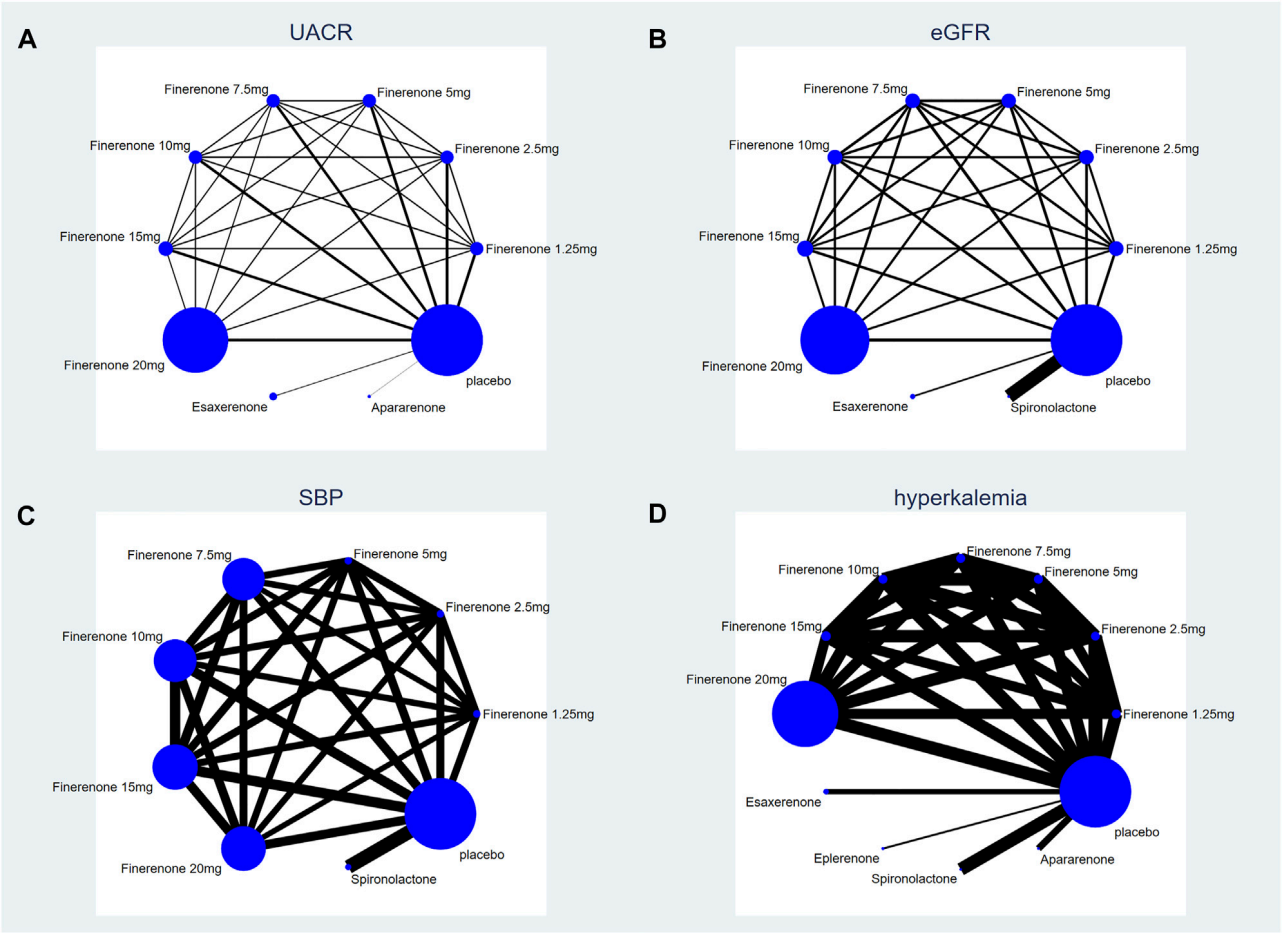
Network plot of treatment comparisons. **(A)** UACR at posttreatment vs. at baseline; **(B)** eGFR at posttreatment vs. at baseline; **(C)** SBP at posttreatment vs. at baseline; **(D)** number of patients suffering from hyperkalemia. The size of the blue nodes is proportional to the number of participants included in the interventions. Interventions are shown by lines, whose thickness represents the number of trials included in our study).

**FIGURE 4:**
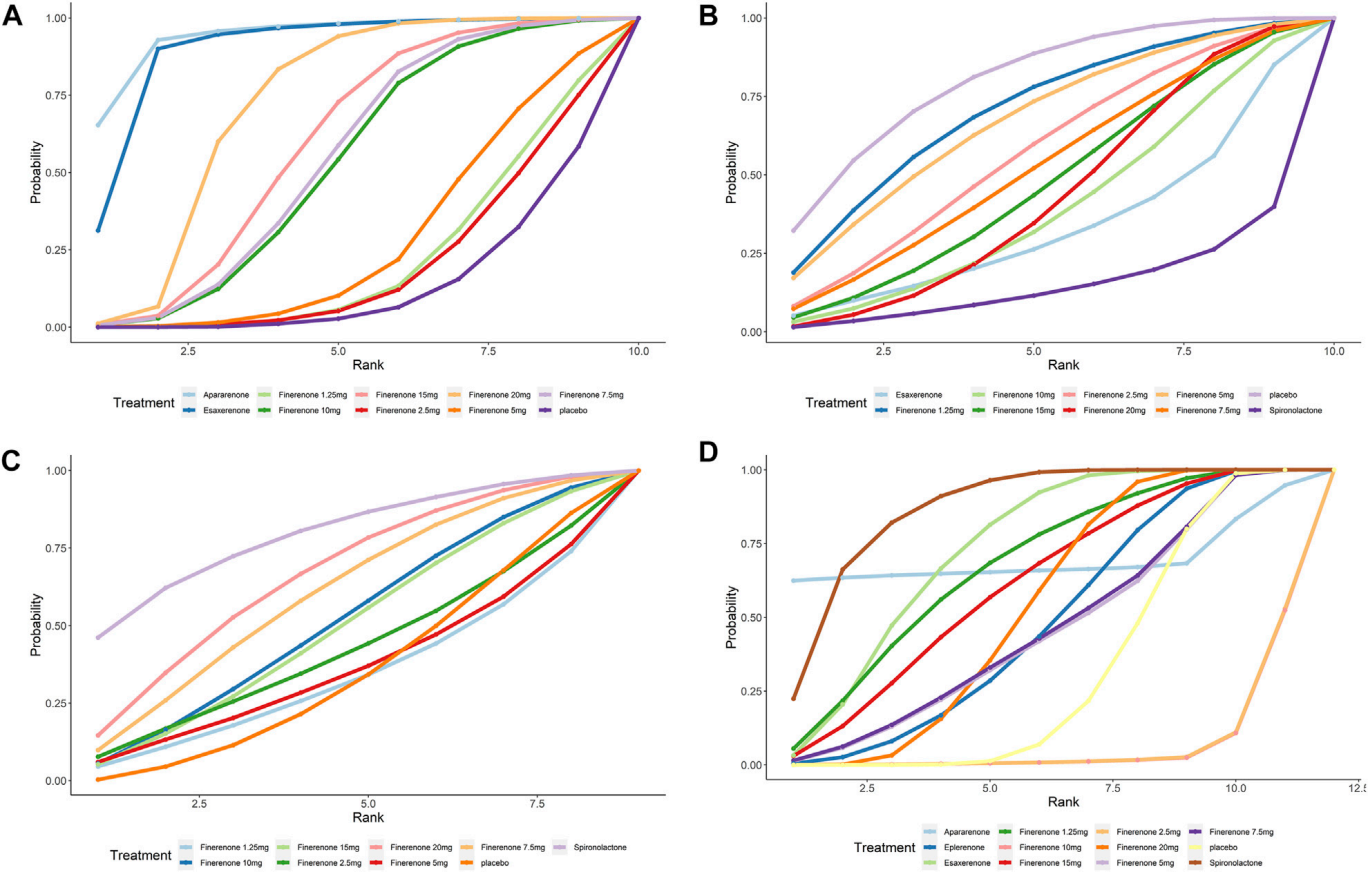
SUCRA probabilities for the effectiveness and safety outcomes of interventions. **(A)** UACR at posttreatment vs. at baseline; **(B)** eGFR at posttreatment vs. at baseline; **(C)** SBP at posttreatment vs. at baseline; **(D)** number of patients suffering from hyperkalemia. A higher SUCRA indicates a higher probability that the drug can reach the endpoint. For example, a higher SUCRA in 5A indicates that the drug has a better effect on UACR decrease).

According to Figure 2, most studies included used randomized grouping methods and applied at least double-blind methods during the treatment. The risk of bias was mainly caused by blinding methods and other biases. Moreover, some studies only provided their results in graphs rather than specific data, so we were incapable of adding their results into our analysis. In Figure 3, the network plot of the Bayesian NMA, nine interventions were reported in the changes of UACR and eGFR at posttreatment vs. at baseline, eight interventions in the change of SBP at posttreatment vs. at baseline, and eleven in hyperkalemia. The detailed SUCRAs of efficacy and safety outcomes was shown in Table 2. The results of the network meta-analysis were shown in Tables 3–6. Figure 4 reports the SUCRA figure of each outcome, and the heterogeneity plot is shown in Supplementary Figure S1.

**TABLE 2:** SUCRAs of treatments according to efficacy and safety outcomes.

	1.25 mg finerenone	2.5 mg finerenone	5 mg finerenone	7.5 mg finerenone	10 mg finerenone	15 mg finerenone	20 mg finerenone	esaxerenone	eplerenone	Spironolactone	Apararenone
UACR	21.17	19.41	26.86	53.43	51.69	58.69	71.16	89.04	—	—	95.88
eGFR	69.92	56.37	66.72	51.82	39.01	46.56	46.48	32.67	—	14.68	—
SBP	33.55	41.68	35.96	59.81	50.66	48.88	65.76	—	—	79.19	—
Hyperkalemia	68.53	6.58	46.36	47.33	6.36	61.31	53.48	73.23	48.13	86.94	69.36

**TABLE 3:** Network meta-analysis of UACR (lower left).

Finerenone 1.25 mg									
0.99 (0.75, 1.3)	Finerenone 2.5 mg								
1.03 (0.78, 1.36)	1.04 (0.79, 1.37)	Finerenone 5 mg							
1.18 (0.91, 1.53)	1.19 (0.92, 1.55)	1.14 (0.89, 1.48)	Finerenone 7.5 mg						
1.17 (0.9, 1.52)	1.18 (0.9, 1.54)	1.14 (0.87, 1.47)	0.99 (0.77, 1.27)	Finerenone 10 mg					
1.21 (0.92, 1.54)	1.23 (0.93, 1.57)	1.18 (0.9, 1.5)	1.03 (0.79, 1.29)	1.04 (0.8, 1.32)	Finerenone 15 mg				
1.29 (1.01, 1.65)	1.31 (1.02, 1.67)	1.26 (0.98, 1.6)	1.1 (0.87, 1.37)	1.11 (0.88, 1.4)	1.07 (0.86, 1.36)	Finerenone 20 mg			
1.62 (1.1, 2.26)	1.64 (1.11, 2.29)	1.57 (1.07, 2.19)	1.38 (0.93, 1.9)	1.39 (0.95, 1.94)	1.34 (0.94, 1.86)	1.25 (0.91, 1.64)	Esaxerenone		
1.78 (1.18, 2.62)	1.8 (1.2, 2.64)	1.72 (1.15, 2.55)	1.51 (1, 2.2)	1.52 (1.02, 2.24)	1.46 (1, 2.18)	1.37 (0.97, 1.92)	1.09 (0.8, 1.58)	Apararenone	
0.95 (0.7, 1.26)	0.96 (0.71, 1.28)	0.92 (0.69, 1.22)	0.81 (0.6, 1.06)	0.81 (0.61, 1.07)	0.78 (0.6, 1.03)	0.73 (0.59, 0.89)	0.59 (0.48, 0.74)	0.53 (0.41, 0.7)	placebo

**TABLE 4:** Network meta-analysis of eGFR (lower left).

Finerenone 1.25 mg									
2.44 (0, 5,027.18)	Finerenone 2.5 mg								
1.2 (0, 2,325.71)	0.5 (0, 1,101.05)	Finerenone 5 mg							
3.27 (0, 6,952.97)	1.33 (0, 3,267.24)	2.69 (0, 6,273.5)	Finerenone 7.5 mg						
7.35 (0, 16,355.21)	3.1 (0, 6,306.9)	5.96 (0, 13,186.77)	2.28 (0, 7,065.87)	Finerenone 10 mg					
4.53 (0, 10,182.99)	1.88 (0, 3,976.36)	3.7 (0, 9,554.33)	1.35 (0, 3,320.35)	0.61 (0, 1,425.72)	Finerenone 15 mg				
5.69 (0, 7,004.93)	2.31 (0, 3,046.62)	4.66 (0, 6,051.47)	1.71 (0, 2,364.52)	0.78 (0, 926.75)	1.26 (0, 1,268.32)	Finerenone 20 mg			
14.53 (0, 520,116.44)	6.06 (0, 197,478.6)	11.47 (0, 550,938.04)	4.49 (0, 212,097.79)	1.94 (0, 76,190.83)	3.19 (0, 118,525.24)	2.58 (0, 26,873.07)	Esaxerenone		
212.81 (0.01, 1,714,689.05)	89.78 (0, 779,941.88)	169.75 (0.01, 1,791,763.97)	64.84 (0, 609,453.8)	28.03 (0, 249,203.46)	49.68 (0, 373,124.62)	36.92 (0, 111,704.93)	14.25 (0, 159,865.75)	Spironolactone	
0.54 (0, 868.12)	0.23 (0, 305.37)	0.44 (0, 840.79)	0.17 (0, 272.31)	0.07 (0, 106.41)	0.12 (0, 149.02)	0.09 (0, 24.82)	0.04 (0, 93.71)	0 (0, 3.8)	Placebo

**TABLE 5:** Network meta-analysis of SBP (lower left).

Finerenone 1.25 mg								
30.61 (0, 3.69e+20)	Finerenone 2.5 mg							
2.65 (0, 2.97e+19)	0.08 (0, 3.21e+17)	Finerenone 5 mg						
3.31e+4 (0, 1.29e+21)	1,032.82 (0, 2.59e+19)	12,053.12 (0, 1.89e+21)	Finerenone 7.5 mg					
1,465.01 (0, 6.84e+19)	39.35 (0, 2.79e+18)	465.79 (0, 4.98e+19)	0.04 (0, 9.22e+11)	Finerenone 10 mg				
712.53 (0, 3.85e+19)	25.26 (0, 1.36e+18)	273.72 (0, 3.45e+19)	0.02 (0, 2.52e+11)	0.61 (0, 8.13e+12)	Finerenone 15 mg			
2.83e+5 (0, 1.36e+22)	8661.49 (0, 3.73e+20)	97,026.08 (0, 1.23e+22)	8.75 (0, 1.05e+14)	215.6 (0, 1.80e+15)	394.28 (0, 8.60e+15)	Finerenone 20 mg		
5.17e+8 (0, 1.35e+28)	1.47e+7 (0, 1.14e+27)	1.77e+8 (0, 2.07e+28)	13,591.8 (0, 1.167e+21)	337,539.68 (0, 3.49e+22)	6.24e+05 (0, 6.16e+22)	1,656.86 (0, 1.36e+20)	Spironolactone	
8.28 (0, 2.53e+17)	0.22 (0, 114e+16)	3 (0, 2.69e+17)	0 (0, 1.58e+9)	0.01 (0, 9.50e+10)	0.01 (0, 1.48e+11)	0 (0, 4.51e+08)	0 (0, 603.71)	Placebo

**TABLE 6:** Network meta-analysis of hyperkalemia (lower left).

Finerenone 1.25 mg											
1.37e+9 (4.45, 8.69e+28)	Finerenone 2.5 mg										
2.57 (0.22, 84.84)	0 (0, 0.73)	Finerenone 5 mg									
2.5 (0.2, 81.41)	0 (0, 0.69)	0.96 (0.02, 39.89)	Finerenone 7.5 mg								
1.61e+9 (4.85, 1.89e+29)	1.33 (0, 2.08e+22)	5.76e+8 (1.42, 5.58e+28)	5.76e+8 (1.38, 5.91e+28)	Finerenone 10 mg							
1.32 (0.14, 12.45)	0 (0, 0.29)	0.5 (0.01, 6.53)	0.52 (0.02, 6.93)	0 (0, 0.28)	Finerenone 15 mg						
1.78 (0.19, 16.8)	0 (0, 0.37)	0.69 (0.02, 8.72)	0.71 (0.02, 9.34)	0 (0, 0.35)	1.35 (0.14, 12.84)	Finerenone 20 mg					
0.88 (0.08, 9.5)	0 (0, 0.19)	0.33 (0.01, 4.97)	0.34 (0.01, 5.24)	0 (0, 0.18)	0.66 (0.06, 7.13)	0.5 (0.18, 1.14)	Esaxerenone				
2.18 (0.17, 26.32)	0 (0, 0.48)	0.81 (0.02, 13.07)	0.84 (0.02, 14.79)	0 (0, 0.46)	1.64 (0.13, 19.88)	1.24 (0.33, 3.56)	2.49 (0.55, 10.4)	Eplerenone			
0.41 (0.03, 4.89)	0 (0, 0.09)	0.15 (0, 2.54)	0.16 (0, 2.72)	0 (0, 0.08)	0.31 (0.02, 3.75)	0.24 (0.06, 0.64)	0.47 (0.09, 1.94)	0.19 (0.03, 0.98)	Spironolactone		
0 (0, 1.08e+14)	0 (0, 3.78e+7)	0 (0, 3.64e+13)	0 (0, 3.84e+13)	0 (0, 4.79e+7)	0 (0, 8.70e+13)	0 (0, 6.46e+13)	0 (0, 1.74e+14)	0 (0, 5.59e+13)	0 (0, 3.09e+14)	Apararenone	
3.63 (0.39, 34.29)	0 (0, 0.77)	1.4 (0.04, 17.86)	1.45 (0.04, 18.98)	0 (0, 0.71)	2.75 (0.29, 26.11)	2.03 (1.82, 2.27)	4.07 (1.8, 10.89)	1.65 (0.57, 6.03)	8.49 (3.2, 35.66)	1.63e+4 (0, 1.92e+26)	Placebo

### 3.2 Efficacy outcomes

#### 3.2.1 Ratio of urine albumin creatine ratio at posttreatment vs. at baseline

**FIGURE 5:**
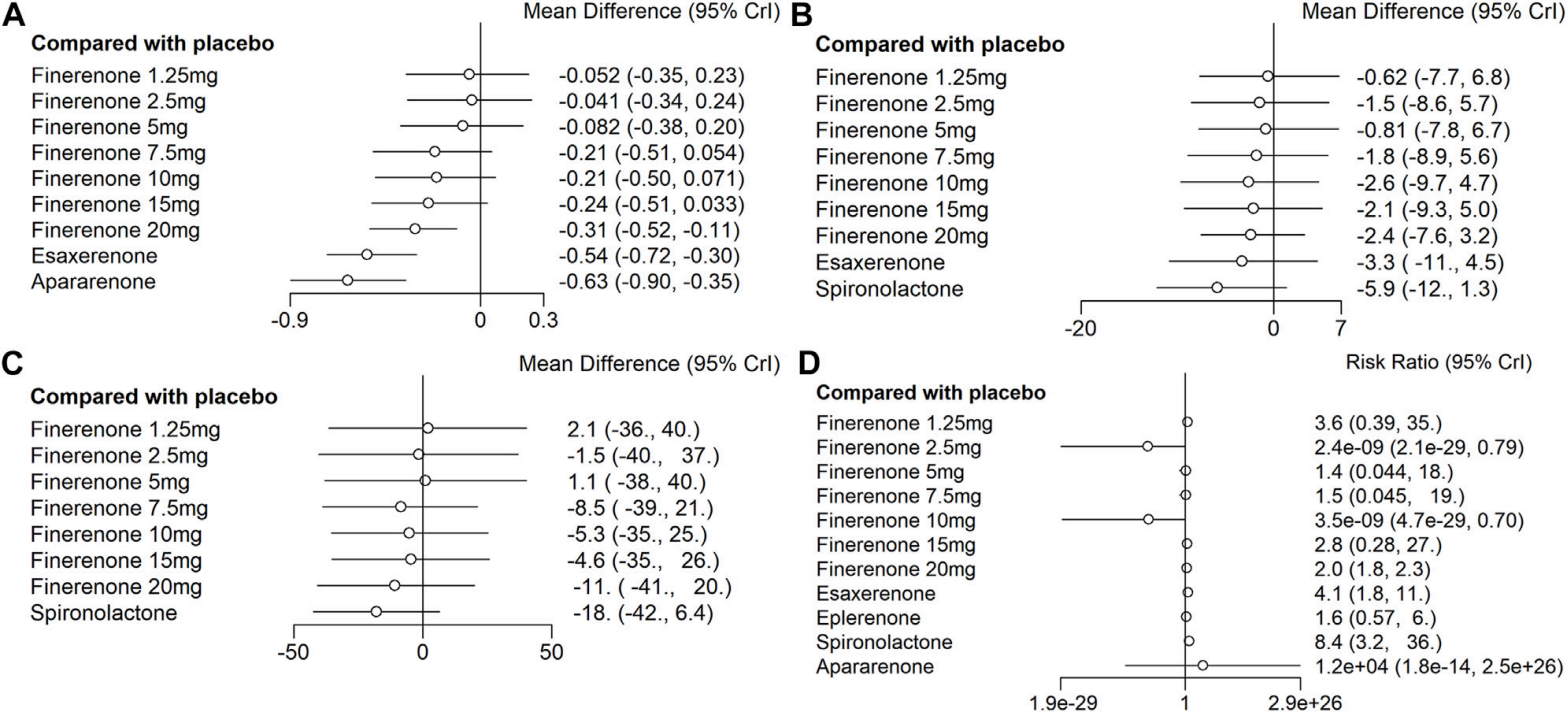
Data of network comparison between placebo and MRAs. **(A)** UACR at posttreatment vs. at baseline; **(B)** eGFR at posttreatment vs. at baseline; **(C)** SBP at posttreatment vs. at baseline; **(D)** number of patients suffering from hyperkalemia).

Compared with placebo, the efficacy of finerenone in reducing albuminuria was observed to be dose-dependent, because a high dose of finerenone could effectively reduce UACR in DKD patients (MD −0.31, 95% CI: −0.52, −0.11), while apararenone (MD −0.63, 95% CI: −0.90, −0.35) and esaxerenone (MD −0.54, 95% CI: −0.72, −0.30) significantly remised proteinuria in patients with DKD. The results of the network meta-analysis are shown in Figure 5A. Ranking all treatments reported ratio of UACR at posttreatment vs. at baseline, we found that apararenone was superior in reducing proteinuria in patients with DKD (SUCRA 95.88%), followed by esaxerenone (SUCRA 89.04%) and 20 mg of finerenone (SUCRA 71.16%) (Figure 4A).

#### 3.2.2 Change in posttreatment estimated glomerular filtration vs. baseline

We found that all treatments were similar in eGFR change since compared with placebo, none of the drugs showed significant changes in eGFR (Figure 5B). Ranking all treatments that reported changes in eGFR at posttreatment vs. at baseline, we found that 1.25 mg of finerenone was superior in maintaining kidney function in patients with DKD (SUCRA 69.92%), followed by 5 mg of finerenone (SUCRA 66.72%), 2.5 mg of finerenone (SUCRA 56.37%), 7.5 mg of finerenone (SUCRA 51.82%), 15 mg of finerenone (SUCRA 46.56%), 20 mg of finerenone (SUCRA 46.48%), 10 mg of finerenone (SUCRA 39.01%), and esaxerenone (SUCRA 32.67%), while spironolactone had the lowest possibility of maintaining kidney function (SUCRA 14.68%) (Figure 4B).

#### 3.2.3 Change in posttreatment SBP vs. baseline

We found that none of the treatments showed significant changes in SBP (Figure 5C). Ranking all treatments that reported changes in SBP at posttreatment vs. at baseline, we found that spironolactone was superior in reducing SBP (SUCRA 79.19%), followed by 20 mg of finerenone (SUCRA 65.76%), 7.5 mg of finerenone (SUCRA 59.81%), 10 mg of finerenone (SUCRA 50.66%), 15 mg of finerenone (SUCRA 48.88%), 2.5 mg of finerenone (SUCRA 41.68%), 5 mg of finerenone (SUCRA 35.96%), and 1.25 mg of finerenone (SUCRA 33.55%) (Figure 4C).

### 3.3 Safety outcomes

We found that spironolactone (RR 8.4, 95% CI 3.2, 36.0), esaxerenone (RR 4.1, 95% CI 1.8, 11.0), and 20 mg of finerenone (RR 2.0, 95% CI 1.8, 2.3) had significant risks of increasing the morbidity of hyperkalemia (Figure 5D). Ranking all treatments reported morbidity of hyperkalemia at posttreatment vs. at baseline, we found that 10 mg of finerenone was associated with the lowest possibility of leading to hyperkalemia among all drugs (SUCRA 6.36%), followed by 2.5 mg of finerenone (SUCRA 6.58%), 5 mg of finerenone (SUCRA 46.36%), and spironolactone was associated with the highest possibility of increasing the morbidity of hyperkalemia (SUCRA 86.94%) (Figure 4D).

## 4 Discussion

Although MRAs have been proven effective in decreasing albuminuria in addition to RAAS blockers (Lozano-Maneiro and Puente-Garcia, 2015; Chung et al., 2020), few head-to-head studies have been performed to assess the efficacy of individual MRAs. In addition, previous meta-analyses related to MRAs focused on either a specific kind of MRA (Zhao et al., 2016; Zheng et al., 2022); or different classes of drugs (Elliott and Meyer, 2007), so the existing evidence of MRAs treating DKD is limited. This study suggested the priority of several new nonsteroidal MRAs in reducing albuminuria. None of the treatments included was associated with significant risks of worsening kidney function. The efficacy of MRAs in controlling the systolic blood pressure of DKD patients remains unclear. Meanwhile, spironolactone, esaxerenone, and 20 mg of finerenone demonstrated higher morbidity of hyperkalemia in patients suffering from chronic kidney disease and T2DM.

Our network meta-analysis indicated that several new non-steroidal mineralocorticoid receptor antagonists, including apararenone, esaxerenone, and 20 mg of finerenone, could significantly reduce the UACR in patients with DKD, which is consistent with a recent meta-analysis indicating that finerenone has an optimal effect on albuminuria in patients with DKD (Zheng et al., 2022). While apararenone is still in clinical trials, and esaxerenone is used for hypertension treatment, more trials are needed to systematically evaluate the renoprotection efficacy of esaxerenone and apararenone in patients with DKD. Compared with esaxerenone and apararenone, 20 mg of finerenone included more evidence from thousands of participants, which presented a more comprehensive reflection of its effect.

Both the results of our NMA and ranking SUCRAs revealed that most included MRA could not significantly impact kidney functions, while finerenone was associated with potential priority in protecting kidney function and spironolactone was in connection to the decrease of eGFR, according to the ranking SUCRA, which was consistent with another meta-analysis published before (Pei et al., 2018). Chung et al. (2020) reported that spironolactone significantly decreased eGFR in patients with chronic kidney disease with or without diabetes. For several nonsteroid MRAs, however, eGFR decreased after the administration of MRAs and returned to baseline during the follow-up period, which suggested that no severe damage was caused by drug administration (Ito et al., 2019; Wada et al., 2021). The decrease in eGFR reduction in spironolactone shown in Figure 4 might be related to the acute kidney injury caused by steroidal MRAs, and the potential priority of finerenone in NMA revealed greater benefits of finerenone in kidney protection with no dose-dependency observed (Barrera-Chimal et al., 2022). The possible reasons for the different conclusions about spironolactone in impact on kidney function may be the heterogeneity of eligible studies such as different durations of studies, and different baseline information of patients.

Regarding controlling blood pressure, no significant superiority was observed in spironolactone or finerenone compared with placebo; however, spironolactone may have greater priority in controlling blood pressure compared with finerenone, according to the SUCRAs. As a steroid MRA, spironolactone is known for its antihypertensive efficacy by crossing the blood-brain barrier, binding to mineralocorticoid receptors in the brain, and inhibiting the excitation of central sympathetic activity, thus lowering blood pressure (Gomez-Sanchez and Gomez-Sanchez, 2012). A meta-analysis indicated that for patients with resistant hypertension, spironolactone could effectively control both systolic and diastolic blood pressure (Zhao et al., 2017). Moreover, Lin et al. (2021) showed that spironolactone could reduce the hypertensive situation of patients with diabetes. Hou et al. (2015) revealed that the add-on therapy of spironolactone could slow down the progression of DKD due to its antihypertensive use. However, in our NMA, spironolactone did not demonstrate significant antihypertensive effects. This may be related to the different baselines of patients; some suffered from hypertension, while others did not. Hence, further studies are needed to determine the antihypertensive efficacy of MRAs in DKD patients. For patients with stage 2 to stage 4 CKD and diabetes, finerenone did not show great blood pressure lowering effect compared with steroidal MRAs. However, phase 3 trials of finerenone demonstrated that finerenone could significantly reduce cardiovascular events in patients with T2DM and CKD (Lerma et al., 2022). Esaxerenone has been proven to have greater efficacy in hypertension therapy than recommended doses of eplerenone. (Ito et al., 2020a). Esaxerenone has been put into clinical use in Japan (Wan et al., 2021). Apararenone is still under clinical trials, and its antihypertensive efficacy should be studied further.

Previous studies have reported hyperkalemia as a common adverse event of MRAs. Our NMA showed that spironolactone, esaxerenone, and 20 mg of finerenone could significantly increase the morbidity of hyperkalemia compared with a placebo. The mechanism between MRAs and hyperkalemia has already been revealed. Aldosterone binds to receptors in renal collecting tubules, which can increase epithelial sodium channels (ENaC) and K + -Na + -ATPase to promote reabsorption of Na + as well as the secretion of K+ (Rico-Mesa et al., 2020). MRAs obstruct aldosterone by competitively binding to mineralocorticoid receptors, thus raising serum potassium levels and even leading to hyperkalemia. Previous studies have revealed that spironolactone and eplerenone have a higher proportion of mineralocorticoid receptors in the kidney, while finerenone has less (Kolkhof and Borden, 2012). Therefore, the morbidity of hyperkalemia in patients administered finerenone should be lower. Hou et al. (2015) indicated that spironolactone could significantly increase the serum potassium level in patients with DKD , which is consistent with our study. Zuo and Xu (2019) revealed that for DKD patients, finerenone had a lower risk of causing hyperkalemia than eplerenone and spironolactone. Eplerenone was used for DKD in various animal experiments and was proven to be effective in decreasing proteinuria (Vodosek et al., 2021). Few studies have been published using eplerenone in DKD patients, although with little increase in serum potassium levels, eplerenone has been banned for treating patients with albuminuria and type 2 diabetes for arterial hypertension (Kloner, 2003). The results of our study suggested that only some doses of finerenone were associated with lower risks of hyperkalemia than those administered eplerenone. The possible reasons may be that the clinical evidence of eplerenone treating DKD was limited, while four studies applying finerenone were eligible in our study, and the duration of treatments was also longer, so the exploration of finerenone was also more comprehensive. Moreover, one study of esaxerenone indicated that the increase in serum potassium level may be related to the decrease in eGFR or higher baseline serum potassium content (Kintscher et al., 2021). The serum potassium level of patients receiving apararenone for treatment increased significantly in a dose-dependent manner, but no patients discontinued the treatment because of hyperkalemia (Wada et al., 2021). Considering that more clinical evidence is needed for esaxerenone and apararenone (Kintscher et al., 2021), finerenone may be the optimal treatment for DKD in controlling hyperkalemia among various MRAs at present.

Concerning the cardiovascular outcomes of MRAs for DKD patients, we found that spironolactone and eplerenone have been proven effective in treating hypertension, and one meta-analysis showed that in addition to ACEI/ARB, MRA could significantly reduce systolic and diastolic blood pressure in patients with proteinuric CKD. However, due to the high risk of adverse events such as hyperkalemia and gynecomastia, therapy using spironolactone and eplerenone in proteinuric CKD has been largely replaced by better methods (Lytvyn et al., 2019). Compared with placebo, finerenone could significantly reduce the incidence of cardiovascular outcomes, including cardiovascular death, nonfatal stroke, and nonfatal myocardial infarction, with less hyperkalemia risk (Filippatos et al., 2021). In summary, compared with other MRAs, 20 mg of finerenone has been proven effective in improving proteinuria with more supporting evidence, maintained kidney function, superior cardiovascular protection, and acceptable incidence of an adverse event.

Several new drugs and therapies have been developed for the treatment of diabetic kidney disease. Studies have shown that ACE inhibitors are related to alleviating DKD through DPP-4 and TGFβ pathways. (Srivastava et al., 2020). Some patients in the included studies used sodium-glucose cotransporter-2 (SGLT2) inhibitors for glucose-lowering therapy. Neuen et al. (2022) found that SGLT2 inhibitors can reduce the risk of hyperkalemia in patients with T2DM and chronic kidney disease. Moreover, studies have shown that SGLT2 inhibitors have not only renoprotective but also cardioprotective effects in patients with CKD (Sarafidis et al., 2021). Several MRAs have been proven effective in CKD therapy. However, studies combining MRAs with SGLT2 inhibitors are limited. A preclinical study showed that the combination of finerenone and empagliflozin could improve cardiovascular and renal outcomes in a model with hypertension-induced cardiorenal disease (Kolkhof et al., 2021). Rossing et al. (2022) indicated that receiving SGLT2 inhibitors in addition to finerenone was associated with greater UACR improvement, while kidney and cardiovascular outcomes remained consistent whether SGLT2 inhibitors were used. In addition, in an extended analysis of dapagliflozin in the treatment of CKD patients with or without diabetes, we found that the efficacy of dapagliflozin in renal protection remains similar in CKD patients with or without MRAs prescribed before (Provenzano et al., 2022). Interrelated to MR, glucocorticoid receptor (GR) is known as an antifibrotic molecule in the development of DKD. Preclinical studies have indicated that diabetes promotes the development of renal fibrosis in mice lack of GR compared to control mice, which is related to abnormal cytokine and chemokine reprogramming and upgraded Wnt signaling. (Srivastava et al., 2021).

Our study has some limitations. First, the number of comparisons and sample size varied, thus some large-sample studies may influence the overall result more than those with small sample sizes. Second, no outcomes included all 12 trials, which may cause potential bias. Third, varied baseline treatments, such as whether ACEIs/ARBs were used during treatment, significantly impacted the clinical outcomes. Fourth, some kinds of drugs had a small number of treatments included, causing unknown bias or uncertainty. Fifth, hyperkalemia was the only adverse event analyzed. Sixth, no subgroup analysis was performed in this NMA. The seventh, head-to-head comparison was absent in this NMA due to the absence of direct comparisons between different MRAs in patients with DKD. Eighth, due to a lack of data, spironolactone and eplerenone were not included in the superiority analysis of UACR, which can be further explored in the future. In the future, relevant trials can focus on the direct head-to-head comparison administrating different kinds of MRAs for DKD patients. Meanwhile, the mechanism of DKD should be explored further and more new therapies and drugs including glycolysis inhibitors, ROCK isoforms, and SIRT3 still need further study.

This study systematically searched existing RCTs of MRAs on patients with DKD, and we may recommend the use of 20 mg finerenone in DKD treatment compared with other types of MRAs based on existing data. Further head-to-head studies combining SGLT2 inhibitors with different MRAs may support this conclusion.

## Data Availability

The original contributions presented in the study are included in the article/Supplementary Material, further inquiries can be directed to the corresponding author.
